# Porcine Beclin1 facilitates the proliferation of porcine circovirus type 2 by augmenting autophagy in PK-15 cells

**DOI:** 10.3389/fcimb.2026.1674170

**Published:** 2026-04-30

**Authors:** Yiting Li, Xin Wu, Jianqiang Han, Juan Geng, Yunyue Hu, Yifang Zhang, Jianling Song, Zhenxing Zhang, Liangyu Yang, Jun Chai, Lei Li

**Affiliations:** 1 College of Veterinary Medicine, Yunnan Agricultural University, Kunming, Yunnan, China; 2 College of Animal Science and Technology, Yuxi Agricultural Vocation - Technical College, Yuxi, Yunnan, China; 3 Yunnan Tropical and Subtropical Animal Virus Disease Laboratory, Yunnan Animal Science and Veterinary Institute, Kunming, China

**Keywords:** autophagy, Beclin1, porcine circovirus type 2, porcine kidney 15 cell line, viral proliferation

## Abstract

Porcine circovirus type 2 (PCV2) is a significant immunosuppressive pathogen that poses a serious threat to the global swine industry. PCV2 infection often triggers secondary infections with multiple pathogens, making it crucial to elucidate its replication mechanisms for precise prevention and control. Numerous studies have demonstrated that autophagy plays a crucial role in viral infection. However, as the core regulator of autophagy, it has remained unclear whether Beclin1 is involved in the replication of PCV2. In this study, we utilized PK-15 cells as a model to investigate the role of Beclin1-mediated autophagy in PCV2 proliferation. The results showed that PCV2 infection significantly induced autophagy. Knockdown of Beclin1 simultaneously suppressed autophagic activity and viral proliferation, whereas overexpression of Beclin1 markedly enhanced autophagy and increased viral proliferation. Pharmacological experiments further confirmed that the autophagy inducer rapamycin amplified Beclin1-mediated autophagy and promoted viral proliferation, whereas the autophagy inhibitor MHY1485 exerted the opposite effect. In summary, this study reveals a novel function of Beclin1 in positively regulating PCV2 proliferation by enhancing autophagic activity in PK-15 cells, providing a theoretical basis for targeting the autophagy pathway to develop strategies for PCV2 control.

## 1 Introduction

Porcine circovirus-associated disease (PCVAD), caused by PCV2, represents one of the most economically significant infectious diseases affecting the global swine industry (Turlewicz-Podbielska et al., 2022). PCV2, a member of the Circoviridae family, is a non-enveloped, icosahedral virus approximately 20 nm in diameter. Its genome consists of a single-stranded, circular DNA molecule of about 1.7 kb, encoding 11 open reading frames (ORFs) (Ren et al., 2016; Gao et al., 2024). Among these, ORF1 encodes the replication-associated proteins Rep and Rep’; ORF2 encodes the major capsid protein (Cap), which carries the principal neutralization epitopes; ORF3 and ORF4 encode pro-apoptotic and anti-apoptotic proteins, respectively, suggesting that the virus finely regulates the host cell’s survival-death balance to create a microenvironment conducive to its own replication (Park et al., 2023).

Autophagy is a highly conserved degradation and recycling system in eukaryotic cells, responsible for clearing misfolded proteins, damaged organelles, and invading pathogens (Parzych and Klionsky, 2014; Zuo et al., 2023). It is classified into microautophagy, macroautophagy (hereafter referred to as autophagy), and chaperone-mediated autophagy based on the substrate delivery mechanism (Zwilling and Reggiori, 2022). Autophagy is orchestrated by more than 30 autophagy-related (Atg) proteins (Zientara-Rytter and Subramani, 2020). Beclin1 (the mammalian homolog of yeast Atg6), a core subunit of the class III phosphatidylinositol 3-kinase (PI3K-III) complex, initiates autophagosome nucleation and is negatively regulated by Bcl-2 family proteins (Liang et al., 2014; Ye et al., 2023). Beyond its roles in embryonic development and tumorigenesis, Beclin1 is often hijacked by numerous viruses to evade autophagic degradation, including Enterovirus 71, Human Immunodeficiency Virus Type 1, influenza A virus, African swine fever virus, foot-and-mouth disease virus, alpha-herpesvirus (HSV-1), and human cytomegalovirus (Orvedahl et al., 2007; Kyei et al., 2009; Gladue et al., 2012; Xiang et al., 2020; Zimmermann et al., 2021; Guo et al., 2023; Sun et al., 2023).

Recent years have seen growing evidence highlighting the proviral role of autophagy during PCV2 infection (Gu et al., 2016; Wu et al., 2025). The host protein DNAJB6 was shown to enhance PCV2 production by promoting autophagosome formation (Han et al., 2020); glutamine deprivation activates the ROS-JAK2/STAT3 axis to induce autophagy, thereby supplying energy and membrane structures for PCV2 replication; under oxidative stress, autophagy indirectly facilitates viral proliferation by suppressing apoptosis (Liu et al., 2018). Additionally, ochratoxin A (OTA) has been reported to enhance PCV2 replication via Beclin1-dependent autophagy (Gan et al., 2018).

Previous studies have demonstrated that PCV2 infection activates the autophagic flux in PK-15 cells, and both pharmacological inhibition of autophagy (using 3-MA) and siRNA-mediated knockdown of Beclin1 reduce viral titers, suggesting that Beclin1 may serve as a critical switch exploited by the virus to harness autophagy (Qian et al., 2017).Although existing studies have suggested an association between Beclin1 and PCV2 replication, whether PCV2 achieves efficient replication by hijacking the Beclin1-mediated autophagic process remains a core question that requires further verification and clarification.

This study focused on the core regulatory axis of “Beclin1-autophagy-PCV2 replication”. By combining gene knockdown/overexpression techniques with pharmacological modulation, we confirmed that Beclin1 is an indispensable host factor for PCV2 replication—it specifically enhanced autophagic activity to create favorable conditions for viral proliferation. These findings not only uncover a new function by which PCV2 exploits the host autophagic pathway for replication but also provide potential insights for developing autophagy-targeted therapeutic strategies against PCVAD.

## 2 Materials and methods

### 2.1 Cells, virus and reagents

Porcine kidney cells (PK-15) and human embryonic kidney cells (HEK293T) were maintained at the Microbiology Laboratory of Yunnan Agricultural University. The cells were cultured in DMEM medium (Gibco, Thermo Fisher Scientific) supplemented with 5% fetal bovine serum (Biological Industries, Israel), 100 U/mL penicillin, and 100 µg/mL streptomycin (Biosharp) at 37°C in a 5% CO_2_ atmosphere. Cell transfection was carried out using LipoRNAi™ transfection reagent (Beyotime Biotechnology Co., Ltd.) following the manufacturer’s protocol.

The prevalent PCV2d strain KM-2018 in Yunnan (GenBank accession number: MK405698) was isolated, identified, and preserved by our research group. Plasmids associated with the lentiviral system, such as the expression vector pCDH-CMV-MCS-EF1-GFP+Puro, the packaging plasmid pMD2.G, and the envelope plasmid psPAX2, were procured from Hunan Fenghui Biotechnology Co., Ltd. Cells were infected with a specified multiplicity of infection (MOI) in serum-free medium. Following a two-hour incubation period, the cells were washed twice with PBS and added with fresh medium containing 2% fetal bovine serum for subsequent experiments.

The Beclin1 Polyclonal antibody was procured from Proteintech, the PCV2 replicase antibody from GeneTex, the LC3B Rabbit mAb and the Phospho-SQSTM1/p62 (Ser403) Rabbit mAb from Selleck Chemicals, and the AF647-labeled Goat Anti-Rabbit IgG (H+L) from Beyotime Biotechnology Co., Ltd.

### 2.2 Plasmid construction

The coding gene of porcine Beclin1 was amplified from PK-15 cell cDNA and subsequently cloned into the pMD19-T vector (TaKaRa Biotechnology (Beijing) Co., Ltd.). Positive clones were identified through blue-white plaque screening and PCR. Following sequencing verification, the resulting recombinant plasmid was designated as pMD19-T-Beclin1.

### 2.3 siRNA and transfection

Specific siRNAs targeting Beclin1 mRNA were designed and synthesized based on the Beclin1 mRNA sequence (GenBank accession number: XM_013980932.2), comprising si-Beclin1-1, si-Beclin1-2, si-Beclin1-3, and a negative control siRNA (si-NC) (**Table 1**). For optimization of transfection conditions, PK-15 cells were seeded at a density of 4×10^5^ cells per well in 12-well plates. Complexes were formed using LipoRNAi™ Transfection Reagent (Beyotime) and Cy3-labeled siRNAs at concentrations ranging from 10 to 100 nM. Transfection efficiency was assessed via fluorescence microscopy after 48 hours to determine the optimal siRNA concentration. In the interference efficiency validation experiment, PK-15 cells were seeded at 2×10^5^ cells per well in 24-well plates and transfected with the identified optimal siRNA concentration. Beclin1 mRNA levels were monitored at 6–48 hours post-transfection. Each experiment was performed with three independent biological replicates, and the data were presented as means. Prior to the infection experiment, cells were cultured at 37°C for 24 hours and subsequently infected with PCV2 for 48 hours.

**Table 1:** The siRNA sequences.

Name	Sequence
si-Beclin1-1	GGAGCUUACAGCUCCAUUAtt
	UAAUGGAGCUGUAAGCUCCtt
si-Beclin1-2	AGGAUGAUGUCUACAGAAAtt
	UUUCUGUAGACAUCAUCCUtt
si-Beclin1-3	CAGUGAAUUUAAAAGACAAtt
	UUGUCUUUUAAAUUCACUGtt

### 2.4 Construction of Beclin1 overexpressed cell line

The recombinant plasmid pCDH-CMV-Beclin1 was generated through molecular cloning methods by ligating the porcine Beclin1 gene into the linearized vector pCDH-CMV-MCS-EF1-GFP+Puro using T4 DNA ligase (TaKaRa Biotechnology (Beijing) Co., Ltd.). Subsequently, the construct was introduced into stb13 competent cells (TransGen Biotech, Beijing). Positive clones were identified through ampicillin (Amp) resistance screening, colony PCR, and double digestion, with sequence accuracy confirmed via Sanger sequencing. Following the extraction of the endotoxin-free recombinant plasmid, it was co-transfected with lentiviral packaging plasmids pMD2.G and psPAX2 into HEK-293T cells to generate lentiviruses utilizing Lipo800TM Transfection Reagent (Beyotime Biotechnology Co., Ltd.).

The concentrated viral solution was utilized to infect PK-15 cells, leading to the establishment of stable cell lines, namely PK-15-Beclin1 and the control PK-15-pCDH, achieved through puromycin selection at an optimal concentration of 5 μg/mL (Solarbio Life Sciences, Shanghai). The expression of Beclin1 mRNA was assessed via quantitative real-time PCR (qPCR) with β-actin as the internal reference, and data analysis was conducted using the 2-ΔΔCt method. The overexpression of Beclin1 was subsequently validated through Western blotting employing an anti-Beclin1 antibody. Throughout the study, appropriate controls including empty vector and untransfected samples were included, and all procedures were executed following standardized protocols and under aseptic conditions. The experiment was conducted with three independent biological replicates.

### 2.5 Real-time quantitative PCR analysis

Total cellular RNA was extracted using RNAiso Plus reagent (Takara) according to the manufacturer's instructions. Complementary DNA (cDNA) was synthesized from RNA samples using the HEvo M-MLV RT-PCR Kit (Hunan Accurate Biotechnology Co., Ltd.). PCV2 DNA was extracted with the SteadyPure Viral DNA/RNA Extraction Kit (Hunan Accurate Biotechnology Co., Ltd.) following the protocol provided by the manufacturer.

Quantitative PCR(qPCR) was performed on a StepOnePlus Real-Time PCR System (Thermo Fisher Scientific, USA) using the SYBR Green Pro Taq HS Premixed qPCR Kit III (Hunan Accurate Biotechnology Co., Ltd.). The transcriptional level of porcine Beclin1 (pBeclin1) mRNA was normalized to porcine β-actin as the internal reference gene. Relative expression fold changes of pBeclin1 mRNA in PK-15 cells either infected with PCV2 or mock-infected were calculated using the 2-ΔΔCt method at different time points. The primer sequences used in this study are listed in **Table 2**. All primers were synthesized by Kunming Qingke Biotechnology Co., Ltd.All experiments were performed in three independent biological replicates, and data were presented as the mean values.

**Table 2:** Primers information.

Gene product	Sense primer (5’ to 3’)	Antisense primer (5’ to 3’)
PCV2	ACCGTTACCGCTGGAGAAGGAAAAA	TGGTTACACGGATATTGTAGTCCTG
Beclin1	ACCTCCATAGAAGATTCTAGAATGGAGGGGTCTAAGACATCCA	GATCGCAGATCCTTCGCGGCCGCTCATTTGTTATAAAACTGTGAGGATACC
qBeclin1	ATGGAGGGGTCTAAGACATCC	TCATTTGTTATAAAACTGTGAGGATAC
qPCV2	ATCTTCAACACCCGCCTCT	CAGGGCCAGAATTCAACCTT
β-actin	CTGTCCCTGTATGCCTCTG	ATGTCACGCACGATTTCC

The underscores were the sites for restriction digest.

### 2.6 Indirect immunofluorescence assay (IFA)

PK-15 cells and their stable transfectants (PK-15-Beclin1, PK-15-pCDH) were seeded at a density of 1×10^6^ cells per well in 6-well plates and maintained at 37°C with 5% CO_2_ until reaching 30-50% confluency. Subsequently, cells were transfected with either si-Beclin1–1 or si-NC, followed by a 24-hour incubation period for the transfected groups. Upon reaching 70-80% confluency in all experimental groups, cells were exposed to a PCV2 virus solution (non-inoculated cells were used as controls). Following a 2-hour virus adsorption period, the maintenance medium was replenished, and cells were cultured for an additional 48 hours.

Cells were fixed in 4% paraformaldehyde for 20 minutes and permeabilized with 0.1% Triton X-100 (TaKaRa Biotechnology (Beijing) Co., Ltd.) for 15 minutes. Subsequent procedures involved blocking with 1% BSA at room temperature for 1 hour, incubation with a primary antibody (Porcine circovirus type 2/PCV2 replicase antibody, GeneTex) at a 1:100 dilution overnight at 4°C, and exposure to a fluorescent secondary antibody (CoraLite594-conjugated Goat Anti-Rabbit IgG (H+L), Proteintech) at a 1:500 dilution for 1 hour at 37°C in the absence of light. Following rinsing with PBST, cell nuclei were stained with DAPI for 10 min. (Beyotime Biotechnology Co., Ltd.). Imaging was conducted using a fluorescence microscope, and all experiments were conducted with three biological replicates.

### 2.7 Transmission electron microscopy observation

Total protein was extracted from cells using RIPA buffer containing protease and phosphatase inhibitors. After centrifugation at 12,000 × g for 15 min at 4°C, the protein concentration in the supernatant was quantified by BCA assay. Proteins were denatured in 5× loading buffer, separated by SDS-PAGE (initially at 80 V, then increased to 150 V after the dye front entered the separating gel), and transferred to a PVDF membrane via wet transfer at 110 mA for 90 mins. The membrane was blocked with 5% BSA for 2 h, followed by incubation with specific primary antibodies (1:1000 dilution) overnight at 4°C. After washing with TBST, the membrane was incubated with HRP-conjugated secondary antibodies (diluted 1:3000) for 1 h at room temperature. Protein bands were visualized using an ECL detection system, and band intensities were quantified with ImageJ software to determine relative protein expression levels.

### 2.8 Western blotting

Cells were lysed in RIPA buffer supplemented with protease/phosphatase inhibitors, centrifuged at 12,000×g for 15 min at 4°C, and supernatants collected. Protein concentrations were determined via BCA assay: standards and samples were incubated with 200 μL BCA working solution at 37°C for 30 min, absorbance at 562 nm measured using a microplate reader, and a standard curve constructed for quantification. Samples were mixed with 5× reducing loading buffer (4:1, v/v), boiled for denaturation, and subjected to SDS-PAGE (initial 80 V; 150 V after bromophenol blue exited the stacking gel). Proteins were transferred to PVDF membranes by wet transfer (110 mA, 90 min), blocked with 5% BSA for 2 h at room temperature, then incubated with primary antibodies (1:1000) overnight at 4°C and corresponding secondary antibodies (1:3000) for 1 h at room temperature. Membranes were washed three times with TBST (10 min each) between incubations. Protein bands were visualized by ECL and quantified using ImageJ software to calculate relative expression levels of target proteins.

### 2.9 Cell proliferation assay (CCK-8)

To evaluate the effects of Beclin1 silencing and autophagy modulators on cell viability, cells were treated with Beclin1-specific small interfering RNA (si-Beclin1) to regulate Beclin1 expression, incubated for 24 hours, and a negative control group (si-NC) was set up concurrently. To investigate the role of autophagy modulators, PK-15 cells and the Beclin1-overexpressing cell line (PK-15-Beclin1) were seeded in 96-well plates at a density of 4×10^4^ cells per well. After 24 hours of incubation, the cells were treated with the autophagy inducer rapamycin (10 nM), the autophagy inhibitor MHY1485 (10 μM), or an equal volume of dimethyl sulfoxide (DMSO) as a control.

Cell viability was assessed using the CCK-8 kit (Beyotime Biotechnology Co., Ltd.) at 0, 12, 24, and 48 hours post-treatment: 10 μL of CCK-8 solution was added to each well, followed by an additional 2-hour incubation. Absorbance at 450 nm was measured using a microplate reader (Thermo Fisher Scientific). All experiments were performed with 3 independent biological replicates, and the results are presented as mean ± standard deviation.

### 2.10 Statistical analysis

Three replicates were included in each experiment, and each experiment was independently repeated at least three times. The experimental data are presented as group means ± the standard errors of means (SEMs). All statistical analysis was carried out using Prism 8.0 software (San Diego, USA) with the unpaired two-tailed Student’s t-test. Statistical significance was indicated by asterisks (*P < 0.05; **P < 0.01; ***P < 0.001; ****P < 0.0001; ns, not significant [P > 0.05]).

## 3 Result

### 3.1 PCV2 infection induces autophagy in PK-15 cells

**Figure 1:**
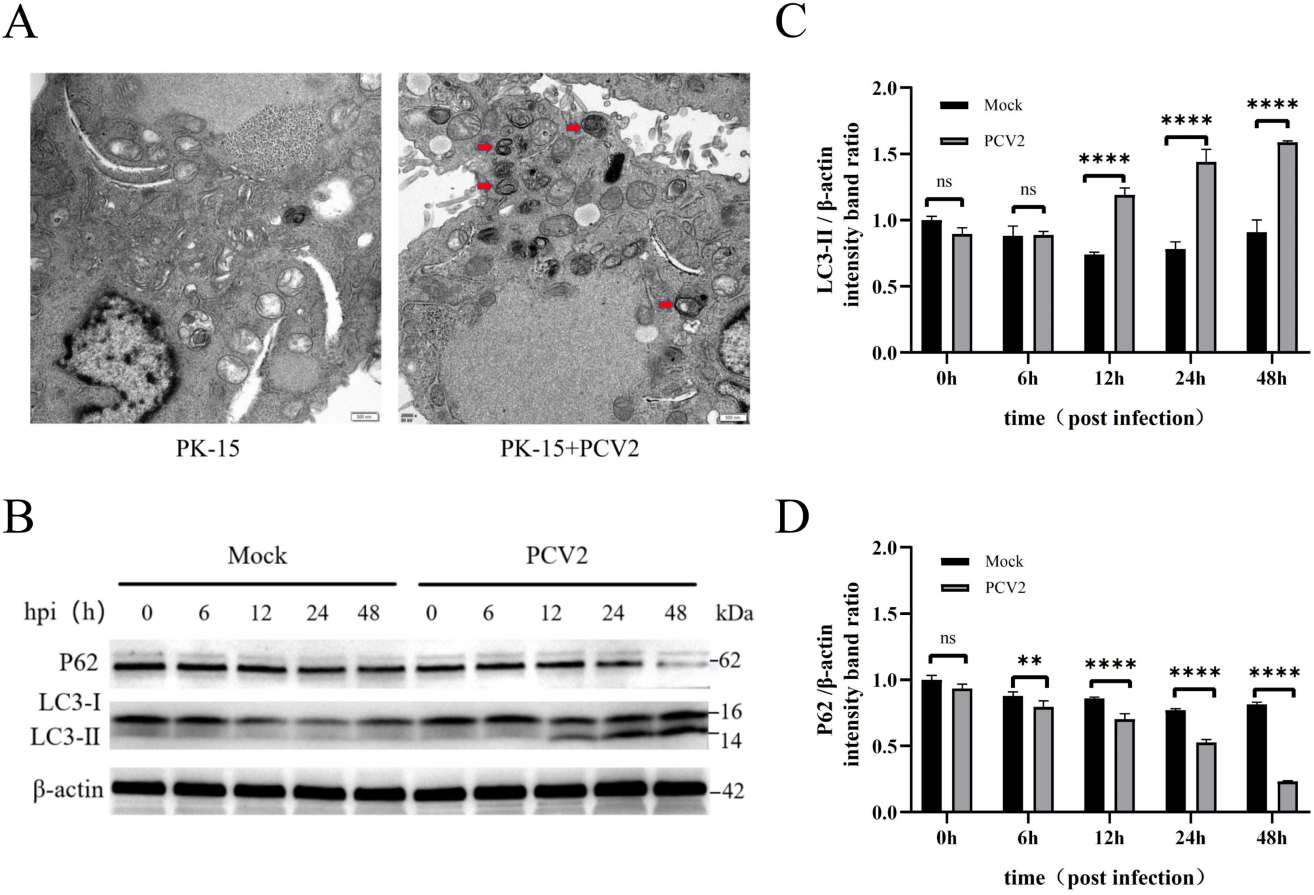
PCV2 infection induces autophagy in PK-15 cells.**(A)** PK-15 cells were divided into two groups: the experimental group was infected with PCV2 at an MOI = 1, and the control group was subjected to mock infection. Both groups were incubated at 37 °C with 5% CO_2_ for 48 hours, and autophagy occurrence was observed by transmission electron microscopy (Note: Red arrows indicate autophagosomes).**(B-D)** PK-15 cells were infected with PCV2 at an MOI = 1, and cell samples were collected at 0, 6, 12, 24, and 48 hours post-infection (hpi).**(B)** Western blot analysis was performed to detect the expression of autophagy-related proteins: Microtubule-associated protein LC3-I/LC3-II and autophagic substrate protein p62. Image J software was used to analyze the grayscale values of LC3-II **(C)** and p62 **(D)** bands in **(B)** for quantitative determination of their relative expression levels. Experiments were independently repeated three times, and data are presented as “mean ± standard deviation”. In statistical analysis, ns indicates no significant difference, while **P < 0.01 and ****P < 0.001 indicate statistically significant differences.

Given Beclin1’s pivotal role in cellular autophagy initiation and progression, our initial focus was to confirm the induction of autophagy during PCV2 infection (**Figure 1**). Transmission electron microscopy (TEM) stands out as a crucial tool for examining autophagic processes. Utilizing TEM, we scrutinized alterations in cellular morphology and ultrastructure in PK-15 cells post-PCV2 infection. Our TEM analysis revealed the absence of discernible autophagosomes in untreated PK-15 cells, contrasting with a notable surge in autophagosome structures evident in PCV2-infected PK-15 cells (**Figure 1A**).

Rabbit anti-LC3 and anti-p62 antibodies were utilized as primary antibodies to assess alterations in the expression of the autophagy marker protein LC3-II/I and the expression level of the autophagy substrate p62, following established protocols (Huang and Liu, 2015; Oh et al., 2017; Yang et al., 2017) (**Figures 1B-D**). It is well documented that upon autophagy induction, LC3-labeled autophagosomes form in the cytoplasm and subsequently merge with endosomes or lysosomes to generate autolysosomes, which create an acidic environment and perform digestive functions within autophagosomes. We evaluated the protein levels of LC3-I, LC3-II, and p62—a widely acknowledged autophagy substrate that interacts with LC3 and is selectively degraded during complete autophagic flux.

The findings indicated that as the duration of PCV2 infection increased, intracellular LC3-I was progressively transformed into LC3-II. The experimental group exhibited a notable increase in LC3-II expression compared to untreated cells (**Figures 1B, C**), alongside a decrease in p62 expression (**Figures 1B, D**). Collectively, these results suggested that PCV2 infection triggered autophagy in PK-15 cells.

### 3.2 Knockdown Beclin1 suppresses autophagy and viral proliferation in PCV2-infected cells

**Figure 2:**
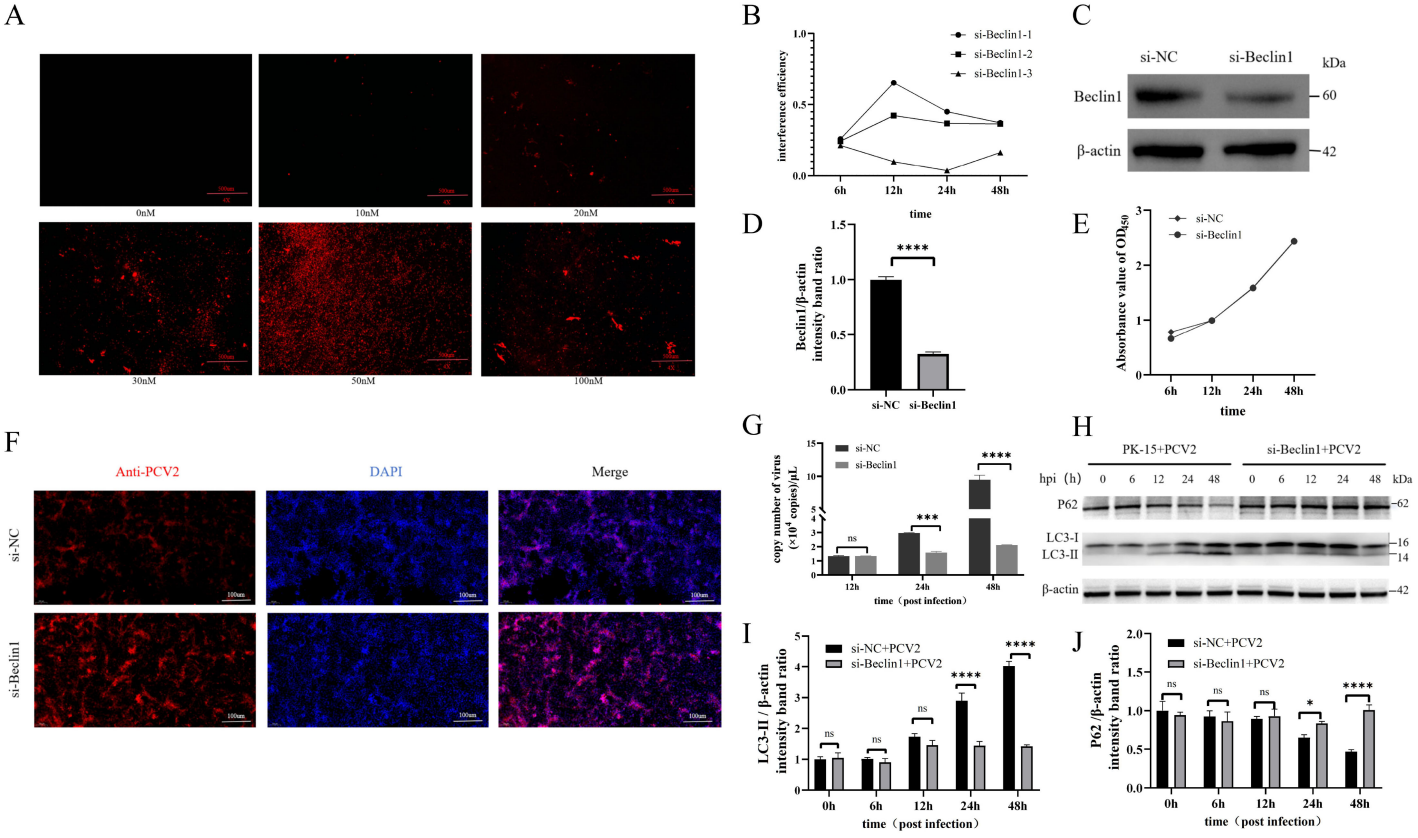
Beclin1 silencing inhibits autophagy activation and viral proliferation in PCV2-infected PK-15 cells. **(A)** PK-15 cells were transfected with 0–100 nM Cy3-labeled Beclin1-siRNA, respectively. The transfection efficiency of siRNA was evaluated by observing Cy3 fluorescence signals under a fluorescence microscope. **(B)** PK-15 cells were transfected with three Beclin1-targeting siRNAs (si-Beclin1-1, si-Beclin1-2, si-Beclin1-3) or negative control siRNA (si-NC), respectively. Cells were harvested at 6, 12, 24, and 48 h post-transfection, and the relative expression level of Beclin1 mRNA was detected via RT-qPCR. si-Beclin1–1 was identified as the optimal siRNA sequence with the highest silencing efficiency (hereinafter referred to as si-Beclin1). **(C, E)** PK-15 cells were transfected with si-Beclin1 (silencing group) or si-NC (control group), respectively. **(C)** The expression level of Beclin1 protein was detected by Western blot analysis; **(D)** ImageJ software was employed to perform grayscale quantitative analysis of Beclin1 protein bands; **(E)** Cell viability was measured using the CCK-8 assay at 6, 12, 24, and 48 h post-transfection to verify the effect of siRNA transfection on cell survival. **(F, G)** After transfection with si-Beclin1 or si-NC, PK-15 cells were infected with PCV2 at an MOI = 1: **(F)** At 48 h post-infection, the status of viral infection was observed using indirect IFA (primary antibody: PCV2 Rep protein-specific antibody); **(G)** Cells were collected at 12, 24, and 48 h post-infection, and the intracellular PCV2 viral genome copy number was quantified via qPCR. **(H-J)** Following transfection with si-Beclin1 or si-NC, PK-15 cell samples were collected at 0, 6, 12, 24, and 48 h post-transfection: **(H)** The expression levels of autophagy-related proteins LC3-I/LC3-II and p62 were determined by Western blot analysis; **(I-J)** Grayscale analysis of LC3-II **(I)** and p62 **(J)** protein bands was conducted using ImageJ software to quantify their relative expression levels. All experiments were independently repeated three times, and data are presented as “mean ± standard deviation”. In statistical analysis, ns indicates no significant difference, while *P < 0.05, ***P < 0.001, and ****P < 0.0001 indicate statistically significant differences.

To investigate the role of Beclin1 in PCV2 infection, we established a Beclin1 knockdown model via siRNA-mediated gene silencing. Based on bioinformatic analysis of the porcine Beclin1 gene, three specific siRNAs and a negative control (si-NC) were designed and screened. Multidimensional validation using fluorescence microscopy identified 50 nM as the optimal transfection concentration (**Figure 2A**). Among these siRNAs, transfection of PK-15 cells with si-Beclin1-1 (hereafter designated as si-Beclin1) for 12 h significantly reduced both Beclin1 mRNA (**Figures 2B**) and protein levels (**Figures 2C, D**) compared to si-NC-transfected PK-15 cells, with CCK-8 assays confirming no cytotoxic effects (**Figure 2E**). Subsequently, PK-15 cells transfected with si-Beclin1–1 or si-NC were inoculated with PCV2 at an MOI of 1. IFA results showed that the proportion of virus-positive cells labeled by red fluorescence was significantly reduced in the si-Beclin1 group (**Figure 2F**). Furthermore, cell samples were collected at 12, 24, and 48 hours post-inoculation (hpi), and quantitative analysis via qPCR confirmed that the viral genome copy number in the si-Beclin1 group was markedly lower than that in the control group at 24 and 48 hpi (**Figure 2G**).To clarify whether this antiviral effect is dependent on autophagic pathway regulation, we detected the expression levels of autophagy-related proteins LC3B and p62 in the two groups of cells at 0, 6, 12, 24, and 48 hpi. In the si-NC control group, PCV2 infection distinctly promoted the conversion of LC3-I to LC3-II (the active form of autophagy) and accelerated the degradation of the autophagy substrate p62 over time (**Figure 2H**). In contrast, the si-Beclin1 knockdown group exhibited a significant reduction in LC3-II accumulation (**Figure 2I**) and an obvious impairment in p62 degradation (**Figure 2J**).

### 3.3 Overexpression of Beclin1 promotes autophagy and viral proliferation in PCV2-infected cells

**Figure 3:**
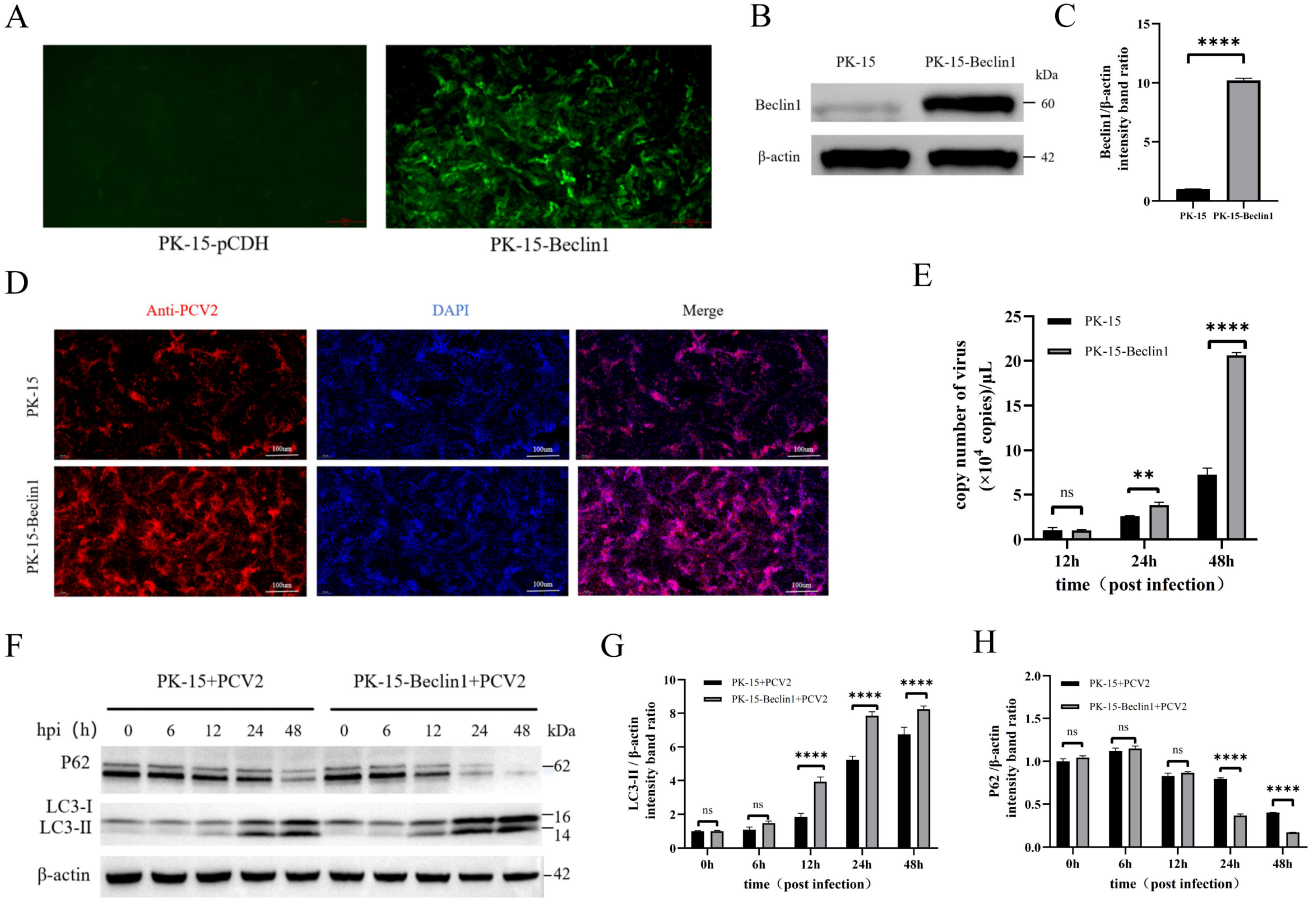
Beclin1 overexpression promotes autophagy activation and viral proliferation in PCV2-infected cells. **(A)** First, HEK-293T cells were transfected with a lentiviral vector expressing Beclin1 to package Beclin1-overexpressing lentiviruses. Subsequently, PK-15 cells were infected with the obtained lentiviruses and serially passaged 10 times to establish a stable Beclin1-overexpressing cell line (PK-15-Beclin1). Fluorescence expression was observed in PK-15-Beclin1 cells and control cells (PK-15-pCDH cells transfected with the empty pCDH vector) under a fluorescence microscope. **(B)** Total proteins were extracted from wild-type PK-15 cells and PK-15-Beclin1 cells. Western blot analysis was performed to detect the expression level of Beclin1 protein, so as to verify the efficiency of stable overexpression. **(C)** ImageJ software was employed to conduct grayscale quantitative analysis of Beclin1 protein bands. **(D, E)** To investigate the effect of Beclin1 overexpression on PCV2 replication: PK-15 cells and PK-15-Beclin1 cells were infected with PCV2 at an MOI = 1, respectively; **(D)** At 48 h post-infection, IFA (primary antibody: PCV2 Rep protein-specific antibody) was used to observe the status of PCV2 infection in the two groups of cells; **(E)** Cells were harvested at 12, 24, and 48 h post-infection, and intracellular PCV2 nucleic acid copy numbers were detected via qPCR to quantify the level of viral replication. **(F-H)** To investigate the effect of Beclin1 overexpression on autophagy in PCV2-infected cells: PK-15 cells and PK-15-Beclin1 cells were infected with PCV2 at MOI = 1, respectively, and samples were collected at 0, 6, 12, 24, and 48 h post-infection; **(F)** Western blot analysis was performed to detect the expression levels of autophagy marker proteins LC3-I/LC3-II and autophagy substrate protein p62; **(G, H)** ImageJ software was used to perform grayscale analysis of LC3-II **(G)** and p62 **(H)** protein bands, respectively, for quantifying their relative expression levels. All experiments were independently repeated three times, and data are presented as “mean ± standard deviation”. In statistical analysis, ns indicates no significant difference, **P < 0.01 and ****P < 0.0001 indicate statistically significant differences.

To further confirm the role of Beclin1 in PCV2-induced autophagy and viral proliferation, we constructed a stable Flag-Beclin1-overexpressed cell line (PK-15-Beclin1) using a lentiviral vector system (**Figures 3A**). Western blotting confirmed a significant upregulation of Beclin1 protein levels (**Figures 3B, C**), indicating successful establishment of the required cell model for functional validation. Subsequently, PK-15 cells (control group) and PK-15-Beclin1 cells (Beclin1-overexpressing group) were synchronously infected with PCV2 at a MOI of 1. IFA showed that the proportion of virus-positive cells labeled with red fluorescence was significantly increased in the PK-15-Beclin1 group at 48 hpi (**Figure 3D**). Furthermore, cell samples were collected at 12, 24, and 48 hpi, and qPCR analysis confirmed that the viral genome copy number in the PK-15-Beclin1 group was markedly higher than that in the control group at 24 and 48 hpi (**Figure 3E**), suggesting that Beclin1 overexpression could effectively facilitate PCV2 proliferation. To clarify whether this pro-viral proliferation effect was mediated by the autophagic pathway, we detected the dynamic changes in expression of autophagy-related proteins LC3B and p62 in the two groups of cells at 0, 6, 12, 24, and 48 hpi (**Figure 3F**). With the progression of infection, a gradual increase in the conversion of LC3-I to LC3-II (the active form of autophagy) and synchronous degradation of the autophagy substrate p62 were observed in the control PK-15 cells. In contrast, the PK-15-Beclin1 overexpressing group exhibited more pronounced LC3-II accumulation (**Figure 3G**) and accelerated p62 degradation (**Figure 3H**).

These results indicated that Beclin1 overexpression could significantly enhance PCV2-induced autophagic flux, implying that it may promote PCV2 proliferation by potentiating the regulation of the autophagic pathway.

### 3.4 Beclin1-dependent autophagy promotes viral proliferation in PCV2-infected cells

**Figure 4:**
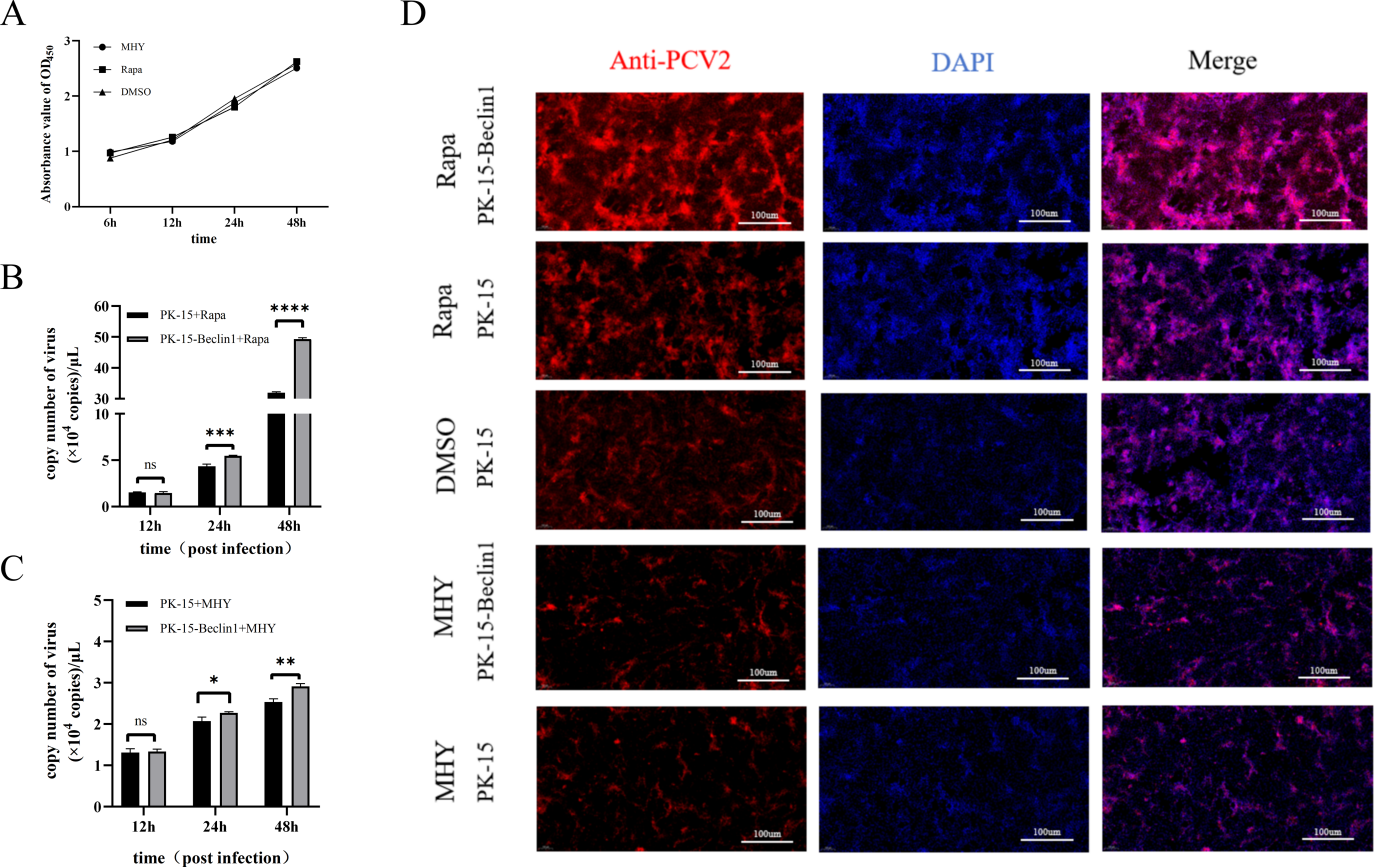
Effects of autophagy modulators on PCV2 replication in PK-15 cells and PK-15-Beclin1 cells. **(A–D)** Wild-type PK-15 cells (PK-15) and Beclin1-overexpressing PK-15-Beclin1 cells were infected with PCV2 following treatment with autophagy modulators: **(A)** Cells were treated with the autophagy activator rapamycin (Rapa) or the autophagy inhibitor MHY1485 (MHY), respectively. At 6 h, 12 h, 24 h, and 48 h post-treatment, cell viability of the two cell lines was evaluated using the CCK-8 assay. **(B)** PK-15 and PK-15-Beclin1 cells were pretreated with Rapa prior to infection with PCV2 (MOI = 1). Cell supernatants were collected at 12 h, 24 h, and 48 h post-infection, and the copy number of PCV2 in the supernatants was quantified by qPCR. **(C)** PK-15 and PK-15-Beclin1 cells were pretreated with MHY1485 prior to infection with PCV2 (MOI = 1). Cell supernatants were collected at 12 h, 24 h, and 48 h post-infection, and the copy number of PCV2 in the supernatants was quantified by qPCR. **(D)** After 24 h of pretreatment with Rapa or MHY1485, both PK-15 and PK-15-Beclin1 cells were infected with PCV2 (MOI = 1). At 48 h post-infection, PCV2 infection was visualized by indirect IFA. All experiments were independently repeated three times, and data are presented as “mean ± standard deviation”. In statistical analysis, ns indicates no significant difference, while *P < 0.05, **P < 0.01, ***P < 0.001, and ****P < 0.0001 indicate statistically significant differences.

To further verify the regulatory role of autophagy in PCV2 infection of PK-15 cells and clarify whether Beclin1 is involved in this regulatory process, we used two autophagy modulators with opposite mechanisms of action for intervention: rapamycin (RAPA) activates autophagy by inhibiting the mTOR signaling pathway, whereas MHY1485 suppresses autophagy by activating the mTOR signaling pathway. Cell viability assays confirmed that neither modulator exerted a significant effect on cell survival at the concentrations used in the experiment (**Figure 4A**). Subsequently, wild-type PK-15 cells and PK-15-Beclin1 cells (Beclin1-overexpressing cells) were pretreated with the above two modulators, respectively, followed by PCV2 infection at a MOI of 1. Quantitative analysis of viral genome copy numbers at different time points (12, 24, and 48 hpi) via qPCR revealed that the PCV2 load in each group increased in a time-dependent manner. In the rapamycin-induced autophagy group, the PCV2 copy number in PK-15-Beclin1 cells at 48 hpi was significantly higher than that in wild-type PK-15 cells, and the viral loads in both groups were markedly higher than those in their corresponding mock-treated groups (solvent only, no modulator added; **Figure 4B**). In contrast, in the MHY1485-treated groups, the PCV2 copy numbers in both wild-type PK-15 cells and PK-15-Beclin1 cells at 48 hpi were significantly lower than those in their respective mock-treated groups, and no statistically significant difference in viral load was observed between the two cell lines (**Figure 4C**). In addition, indirect IFA performed at 48 hpi showed that the intensity of PCV2-specific fluorescence signals was the highest in rapamycin-treated PK-15-Beclin1 cells, whereas the fluorescence signal intensities were significantly attenuated in both MHY1485-treated wild-type PK-15 cells and PK-15-Beclin1 cells (**Figure 4D**).

Collectively, these results demonstrate that autophagy activation can significantly enhance PCV2 proliferation, while autophagy inhibition effectively reduces viral replication efficiency. Moreover, Beclin1 plays a key regulatory role in mediating this autophagy-dependent PCV2 proliferation process.

## 4 Discussion

Autophagy, a critical mechanism for maintaining intracellular homeostasis and combating pathogen infections, involves the key initiating protein Beclin1, which exerts a significant bidirectional effect on virus replication (Sarmadhikari and Asthana, 2025). Previous studies have confirmed that the Beclin1 protein can promote the proliferation of CSFV and Zika virus (ZIKV), while exerting antiviral effects against pathogens such as HSV-1 (Ojha et al., 2019; Rubio and Mohr, 2019; Wang et al., 2022; Sun et al., 2023). Other studies have shown that PCV2 infection can activate the autophagic flux in PK-15 cells; furthermore, pharmacological inhibition of autophagy with 3-MA or Beclin1 gene knockdown mediated by siRNA technology can both significantly decrease the viral titers (Qian et al., 2017). These results suggest that Beclin1 may be a key regulatory factor for PCV2 to complete infection via the autophagic pathway, but there is currently no direct evidence to confirm that PCV2 can hijack the Beclin1 protein of host cells through autophagic action, thereby achieving efficient proliferation of its progeny virus.

To clarify the interaction between PCV2 and host cell autophagy, this study used PK-15 cells as the research object and selected the prevalent PCV2d Yunnan strain KM-2018 for infection experiments. The results were as follows: Electron microscopy observations and protein expression analysis showed that the number of autophagosomes in cells increased significantly at 48 hours post-infection (hpi); meanwhile, the level of the autophagy marker protein LC3-II was significantly elevated at 24 hpi, 36 hpi, and 48 hpi, while the level of the autophagy substrate protein p62 decreased significantly. This is consistent with previous reports that PCV2 possesses the ability to induce host cell autophagy. The above results confirmed that the PCV2d KM-2018 strain could effectively activate the autophagic process in PK-15 cells, laying a solid foundation for subsequent in-depth exploration of the impact of autophagy on PCV2 proliferation.

To determine whether Beclin1 plays a key role in the aforementioned process, further experiments were conducted in this study: After siRNA-mediated Beclin1 knockdown, the proliferation of PCV2 was significantly inhibited at 24 hpi and 48 hpi, and the autophagic phenomenon induced by PCV2 infection (mainly manifested by the increased expression of the autophagy marker protein LC3-II and the degradation of p62) was obviously reversed. In contrast, Beclin1 overexpression significantly promoted PCV2 proliferation and further enhanced PCV2-induced autophagy (mainly characterized by a significant increase in LC3-II expression and enhanced p62 degradation). These results not only emphasize the core function of Beclin1 as a positive regulator of PCV2 replication but also indicated that its role is inseparable from the autophagic process. Notably, this finding stands in sharp contrast to the traditional antiviral function of Beclin1 against ZIKV and HSV-1, but is consistent with its role in promoting CSFV replication through autophagy, highlighting the complexity of Beclin1’s mechanism of action in viral replication—its functional differences depend on the virus species, infected tissues, and infection stage (Karuppan et al., 2020).

Autophagy exerts a dual role in viral infection: it can either act as a host defense mechanism to clear viruses or be hijacked by viruses to promote their own replication. To further clarify the specific impact of autophagy on PCV2 proliferation, this study used the autophagy inducer Rapa and the autophagy inhibitor MHY1485 to regulate the autophagic activity of PK-15 cells and Beclin1-overexpressing cells (PK-15-Beclin1), respectively (Zhao et al., 2016; Cook et al., 2024; Mu et al., 2025). The results showed that compared with the control group, the PCV2 copy number in the Rapa-treated group was significantly increased at 24 hpi and 48 hpi, and the viral copy number in PK-15-Beclin1 cells at 48 hpi was higher than that in PK-15 cells, suggesting that autophagy induction can promote PCV2 replication. Moreover, Beclin1 overexpression may accelerate autophagosome formation through interactions with molecules such as Vps34 and Vps15, providing necessary membrane structures and energy resources for viruses, thereby enhancing this promotional effect (Su et al., 2017; Wang et al., 2024). On the contrary, the PCV2 copy number in the MHY1485-treated group was significantly lower than that in the control group at 48 hpi, and there was no significant difference between PK-15 cells and PK-15-Beclin1 cells, indicating that autophagy inhibition can effectively suppress PCV2 replication and confirming that the promotional effect of Beclin1 on viral replication is dependent on an intact autophagic pathway.

In summary, this study clearly confirmed that PCV2 could induce autophagy in PK-15 cells through the autophagic pathway to enhance its own replication, and Beclin1 plays a key regulatory role in this process. However, the specific molecular mechanism by which Beclin1 regulates PCV2 replication (including its interactions with other autophagy-related proteins and its impact on host cell signaling pathways) still requires further in-depth investigation. In addition, clarifying the interaction between autophagy and the host immune response is of great significance for determining whether autophagy affects PCV2 replication by regulating the immune response. Future research should focus on revealing the molecular mechanism of Beclin1-mediated autophagy in regulating PCV2 replication, as well as the overall role of autophagy in the process of PCV2 infection, so as to provide a theoretical basis for the development of relevant prevention and control strategies.

## 5 Conclusions

This study demonstrated that PCV2 infection could induce autophagy in PK-15 cells and promote its own replication by virtue of this autophagic process. Experimental results showed that after PCV2 infection, the number of autophagosomes in PK-15 cells increased significantly, the expression level of the autophagy marker protein LC3-II increased, while the expression level of the autophagy substrate protein p62 decreased. These results confirmed that PCV2 could effectively activate autophagy in PK-15 cells.

Beclin1, a key regulatory factor of autophagy, plays a core role in the process of PCV2 infection. The results showed that silencing Beclin1 could significantly inhibit PCV2 replication, accompanied by decreased LC3-II expression and p62 protein accumulation; in contrast, overexpression of Beclin1 could promote PCV2 proliferation, with increased LC3-II expression and accelerated p62 protein degradation. Further intervention experiments with autophagy enhancers (such as rapamycin) and inhibitors (such as MHY1485) confirmed that autophagy exerted a positive regulatory effect on PCV2 replication, and Beclin1 played a role in this process. These results indicated that Beclin1 created a favorable intracellular microenvironment for PCV2 replication by regulating the autophagic pathway.

In conclusion, PCV2 enhanced its own replication ability by inducing autophagy in host cells and regulating Beclin1 activity. This finding provides new insights into the pathogenic mechanism of PCV2 and also offers potential targets for the prevention and treatment of PCV2-related diseases. Future studies will further explore the specific molecular mechanism by which Beclin1 regulates PCV2-induced autophagy and the interaction between this process and the host immune response.

## Data Availability

The datasets presented in this study can be found in online repositories. The names of the repository/repositories and accession number(s) can be found in the article/supplementary material.
